# Possible cause of abdominal internal oblique muscle hematoma induced by cough

**DOI:** 10.1002/jgf2.672

**Published:** 2023-12-18

**Authors:** Toshinori Nishizawa

**Affiliations:** ^1^ Department of General Internal Medicine St Luke's International Hospital Tokyo Japan

To the Editor,

I have read with interest the article by Fujimori et al.^1^ This case involves a 40‐year‐old male patient who presented with an abdominal internal oblique muscle hematoma. The intriguing aspect of this case is that the hematoma occurred following a coughing episode, despite the absence of coagulation abnormalities.

Given this unique presentation, it is important to explore all possible underlying factors that could contribute to such an event. One potential cause is the presence of acquired hemophilia, specifically hemorrhagic acquired factor XIII deficiency.^2^ While the patient's platelet count and routine coagulation parameters might appear normal, it is crucial to emphasize that the absence of overt platelet or coagulation abnormalities does not definitively rule out the possibility of acquired factor XIII deficiency.

Acquired factor XIII deficiency is a relatively common disease, but most cases are asymptomatic and do not lead to severe bleeding. However, symptomatic acquired factor XIII deficiency, presenting with hemorrhagic symptoms, is exceedingly rare. This condition can be classified into autoimmune, nonautoimmune, and idiopathic types. Autoimmune acquired factor XIII deficiency is infrequent, with the majority of hemorrhagic acquired factor XIII deficiency being nonautoimmune. Nonautoimmune hemorrhagic acquired factor XIII deficiency, typically presenting as a less severe bleeding disorder, is often attributed to overconsumption or reduced biosynthesis. This can be triggered by various conditions, including disseminated intravascular coagulation, major surgical procedures, liver diseases, and other related disorders. In cases where acquired factor XIII deficiency is suspected, referral to a hematologist is advised, accompanied by a thorough investigation for any underlying pathologies.^2^

Since neither prolonged clotting times nor decreased platelet counts are seen, many cases with unexplained intramuscular and subcutaneous bleeding might be overlooked. However, depending on the site and the amount of bleeding, the bleeding can be fatal, so prompt diagnosis and appropriate treatment are essential. Clinicians should be alert for acquired factor XIII deficiency when seeing such patients and consider measuring the factor XIII activity.

In conclusion, I recommend further investigation into the possibility of acquired factor XIII deficiency in cases similar to the one described. The absence of overt platelet or coagulation abnormalities should not discourage the pursuit of this diagnostic avenue, as this disorder can present with severe bleeding.

## CONFLICT OF INTEREST STATEMENT

The author declares no conflicts of interest for this article.

## NOTATION OF PRIOR ABSTRACT PUBLICATION/PRESENTATION

This work has never been presented.
